# Over-expressing the C_3 _photosynthesis cycle enzyme Sedoheptulose-1-7 Bisphosphatase improves photosynthetic carbon gain and yield under fully open air CO_2 _fumigation (FACE)

**DOI:** 10.1186/1471-2229-11-123

**Published:** 2011-08-31

**Authors:** David M Rosenthal, Anna M Locke, Mahdi Khozaei, Christine A Raines, Stephen P Long, Donald R Ort

**Affiliations:** 1 Global Change and Photosynthesis Research Unit, United States Department of Agriculture, Institute for Genomic Biology, 1206 West Gregory Drive, Urbana, IL, 61801, USA; 2 Department of Plant Biology, Institute for Genomic Biology, 1206 West Gregory Drive, University of Illinois, Urbana, IL, 61801, USA; 3 Department of Biological Sciences, University of Essex, Wivenhoe Park, Colchester, UK, CO43SQ. Current address: Department of Biology, University of Isfahan, Iran; 4 Department of Biological Sciences, University of Essex, Wivenhoe Park, Colchester, CO43SQ, UK; 5 Department of Plant Biology and Crop Sciences, Institute for Genomic Biology, 1206 West Gregory Drive, University of Illinois, Urbana, IL, 61801, USA; 6 Global Change and Photosynthesis Research Unit, United States Department of Agriculture, Institute for Genomic Biology, 1206 West Gregory Drive, Urbana, IL, 61801, USA; Department of Plant Biology and Crop Sciences, University of Illinois, Urbana, IL, 61801, USA

**Keywords:** climate change, photosynthetic carbon reduction cycle, C3 plants, RuBP regeneration, electron transport, improving photosynthesis

## Abstract

**Background:**

Biochemical models predict that photosynthesis in C_3 _plants is most frequently limited by the slower of two processes, the maximum capacity of the enzyme Rubisco to carboxylate RuBP (V_c,max_), or the regeneration of RuBP via electron transport (J). At current atmospheric [CO_2_] levels Rubisco is not saturated; consequently, elevating [CO_2_] increases the velocity of carboxylation and inhibits the competing oxygenation reaction which is also catalyzed by Rubisco. In the future, leaf photosynthesis (*A*) should be increasingly limited by RuBP regeneration, as [CO_2_] is predicted to exceed 550 ppm by 2050. The C_3 _cycle enzyme sedoheptulose-1,7 bisphosphatase (SBPase, EC 3.1.3.17) has been shown to exert strong metabolic control over RuBP regeneration at light saturation.

**Results:**

We tested the hypothesis that tobacco transformed to overexpressing SBPase will exhibit greater stimulation of *A *than wild type (WT) tobacco when grown under field conditions at elevated [CO_2_] (585 ppm) under fully open air fumigation. Growth under elevated [CO_2_] stimulated instantaneous *A *and the diurnal photosynthetic integral (*A*') more in transformants than WT. There was evidence of photosynthetic acclimation to elevated [CO_2_] via downregulation of V_c,max _in both WT and transformants. Nevertheless, greater carbon assimilation and electron transport rates (J and J_max_) for transformants led to greater yield increases than WT at elevated [CO_2_] compared to ambient grown plants.

**Conclusion:**

These results provide proof of concept that increasing content and activity of a single photosynthesis enzyme can enhance carbon assimilation and yield of C_3 _crops grown at [CO_2_] expected by the middle of the 21st century.

## Background

Biochemical models of C_3 _photosynthesis (*A*) predict that *A *is limited by the slowest of three processes: the maximum carboxylation capacity of the enzyme Rubisco (V_c,max_), the regeneration of Ribulose-5-phosphate (RuBP) via whole chain electron transport (J or J_max_), or the inorganic phosphate release from the utilization of triose phosphates (TPU or Pi limited) [1,2]. At current atmospheric [CO_2_], and under non stressed conditions, light saturated *A *operates at the transition between Rubisco and RuBP regeneration limitation. Globally, [CO_2_] is expected to increase from current levels of 390 ppm [3] to over 550 ppm by the middle of this century [4,5]. Elevating [CO_2_] stimulates C_3 _photosynthesis by increasing the substrate for carboxylation, CO_2_, and by reducing photorespiration [6,7]. Therefore, as atmospheric carbon dioxide concentration increases, the control of photosynthesis will shift away from Rubisco limitation toward RuBP regeneration limitation.

Although photosynthetic stimulation at 550 ppm [CO_2_] could in theory increase production by 34%, the observed increase in field C_3 _crops is only 15% [7,8]. Additional future increases in yield potential of the world's major crops through an increase in the proportion of biomass allocated to grain or an increase in the efficiency of light capture will be small, as conventional breeding programs are reaching the theoretical maximum with diminishing returns [9-11]. In contrast, model simulations demonstrate that increasing photosynthetic efficiency under current [CO_2_] by optimizing the biochemistry of photosynthesis could increase the energy conversion efficiency of a given crop in less time than conventional breeding programs [10,12]. At current levels of crop productivity, global food requirements may outpace current crop production by the middle of this century [11,13,14]. Taken together, these observations suggest that direct improvements in photosynthetic efficiency will be needed if we are to meet global food needs in the future.

A common acclimation response of plants grown at elevated [CO_2_] is to allocate fewer resources to Rubisco, thereby downregulating maximum carboxylation capacity (V_cmax_). This so called photosynthetic acclimation makes more resources available for other metabolic processes [6,15]. The implication is that plants could reallocate resources in the photosynthetic carbon reduction (PCR) cycle to increase the efficiency of N use in elevated [CO_2_] [6,7]. In practice, however, plants' photosynthetic resources are not optimally allocated for current [CO_2_] nor is their acclimation response optimal in elevated [CO_2] _[12]. Theoretically, and by reference to a biochemical model of photosynthesis [i.e., [1]], a plant with a 15% decrease in Rubisco content and 15% increase in RuBP regeneration capacity could translate to a 40% increase in *A *and photosynthetic efficiency of nitrogen use at elevated [CO_2_] [Figure 1 in [7]]. It follows that plants engineered with an increased capacity for RuBP regeneration would have a greater increase in productivity in elevated [CO_2_] when compared to wild type plants [16-18].

While 11 enzymes are involved in the PCR cycle, modeling and metabolic control analyses have consistently demonstrated that four enzymes are expected to exert the greatest control of flux in the cycle: ribulose bisphosphate carboxylase-oxygenase (Rubisco), sedoheptulose-1,7-bisphosphatase (SBPase), aldolase and transketolase [19-21]. Two enzymes, Rubisco and SBPase, are predicted to have the greatest control over carbon assimilation [21,22]. Rubisco is well known to be highly abundant, containing 25% of leaf nitrogen (N) [23] and may in some cases account for up to half of leaf N [24]. All attempts to improve photosynthesis by manipulating Rubisco expression, activity, or specificity have yielded poor results, in part because of inherent tradeoffs between activity and specificity of the enzyme and limited capacity to add more of this highly abundant protein [25-27]. An additional hurdle to engineering "better" Rubsico is that the functional enzyme requires the coordinated assembly of eight plastid encoded and eight nuclear encoded subunits to form the large (rbcL) and small (rbcS) units of the hexadecameric enzyme[28,29]. With the exception of Rubisco, the other enzymes exerting the greatest control on photosynthesis all function in the RuBP regeneration portion of the PCR cycle. Thus, near term future improvements in photosynthetic biochemistry in C_3 _plants are more likely to be achieved by improving content or activity of enzymes other than Rubisco [e.g., [18,21,30,31]].

Sedoheptulose-1,7-bisphosphatase (SBPase) is positioned at the branch point between regenerative (RuBP regeneration) and assimilatory (starch and sucrose biosynthesis) portions of the PCR cycle. It functions to catalyze the irreversible dephosphorylation of sedoheptulose1,7-bisphosphate (SBP) to sedoheptulose-7-phosphate (S7P). Transketolase then catalyzes the transfer for a two carbon ketol group from S7P to glyceraldehyde-3-phoshpate (G3P) to yield xylulose-5-phosphate (X5P) or ribose-5-phosphate (R5P) [32]. SBPase is therefore critical for maintaining the balance between the carbon needed for RuBP regeneration and that leaving the cycle for biosynthesis [20].

Previous experiments have demonstrated that tobacco transformants overexpressing SBPase accumulated more biomass than WT in controlled environment chambers at ambient CO_2_[16]. Smaller increases in biomass were reported for mature SBPase overexpressing plants grown in greenhouse conditions [16]. Additionally, overexpression of SBPase in rice did not increase biomass relative to WT for plants grown at ambient CO_2 _levels in two controlled environments [33,34]. The variance in the realized benefit of SBPase overexpression coupled with the fact that RuBP regeneration is highly sensitive to environmental conditions underscores the need to test the response of plants with this single gene manipulation in agronomically relevant conditions [30]. Moreover, models predict that as atmospheric [CO_2_] increases so will the benefit of increasing RuBP regeneration capacity in plants [1,21,35]. Therefore, we compared WT and SBPase overexpressing plants under field conditions at ambient and elevated (ca. 585 ppm) [CO_2_], and we tested the prediction that transformants would exhibit greater stimulation of photosynthesis and yield than WT plants when grown under fully open air CO_2 _fumigation.

## Methods

### Plant Material

Wild type tobacco (*Nicotiana tabacum *L. cv. Samsun) and sense tobacco plants (T_5 _generation *Nicotiana tabacum *L. cv. Samsun) overexpressing a full length *Arabidopsis thaliana *SBPase cDNA, driven by CaMV 35S promoter and the nopaline synthase termination sequence [16], were germinated in Petri dishes and transferred to soil when true leaves emerged. Sense plants (hereafter referred to as 'transformants') were germinated on hygromycin (30 ug/ml) medium. One individual from each of two transgenic lines overexpressing SBPase with varying SBPase levels and several randomly selected wild type (WT) individuals were selected for the experiments. Individuals were subsequently clonally propagated by rooting cuttings in peat pots on misting benches and then planted directly in the field at SoyFACE on July 7 2009.

### SoyFACE site

The SoyFACE facility is located in the Experimental Research Station of the University of Illinois at Urbana-Champaign [36]. Soybean (*Glycine max*) is grown in eight plots (rings 18 meters in diameter) located within a typically managed soybean field of ca. 40 hectares (ha). Four rings are fumigated with pure [CO_2_] and four rings are non-fumigated controls. Six cuttings of each SBPase genotype (11 and 30) and six of WT were planted in subplots within each ring.

Ambient atmospheric [CO_2_] at the beginning of the 2009 field season was ca. 385 ppm and the target [CO_2_] for elevated rings in 2009 was 585 ppm [CO_2_]. In the fumigated rings, 89% of [CO_2_] values recorded every ten minutes from June 19 to September 24, 2009, were within 10% of the target value of 585 ppm. The mean daily [CO_2_] in elevated rings at Soyface during that time was 586.6 ± 19.4 (sd) ppm. Elevated rings were fumigated using a modification of the method of Miglietta *et al*. [37].

### Leaf protein and western blotting

Prior to planting, leaf discs were collected from cuttings and immediately frozen in liquid nitrogen to confirm that sense plants had greater SBPase content than WT. Protein quantifications and western blots were performed following [19]. Sample lanes were loaded on an equal protein basis, separated using 10% (w/v) SDS-PAGE, transferred to polyvinylidene difluoride membrane, and probed using antibodies raised against SBPase and transketolase. Antibody target proteins were detected using horseradish peroxidase conjugated to the secondary antibody and ECL chemiluminescence detection reagent (Amersham, Bucks, UK). Western blots were quantified by densiometry using the molecular imaging Gel Doc XR system (Bio-Rad, Hercules, CA, USA) and imaging software.

### In situ measurements of gas exchange and photosynthetic parameters

The diurnal course of photosynthesis at the SoyFACE site was measured on two young fully expand leaves from each genotype at ambient conditions at both normal (385 ppm) [CO_2_] and elevated (585 ppm) [CO_2_] at five time points on two dates in August, 2009. To ensure that each plant was measured in similar environmental conditions, the LEDs of the controlled environment cuvettes of the gas exchange system (LI-6400, LI-COR, Lincoln, Nebraska) were set to deliver the same ambient light PPFD. Temperature and relative humidity were similarly set to ambient conditions and kept constant for the duration of each measurement period in the diurnal course. To estimate the total daily carbon gain (*A*'), photosynthesis was assumed to increase linearly from 0 μmol CO_2 _m^-2 ^s^-1 ^at dawn (sunrise) to the first measured value and decrease linearly from the last measured values to 0 μmol CO_2 _m^-2 ^s^-1 ^at dusk (sunset). Sunrise and sunset data were determined using the US Naval Observatory website: http://aa.usno.navy.mil/data/docs/RS_OneYear.php. Dew on the leaves prevented us from measuring photosynthesis until about 10:00 h. We estimated *A*' for each block by integration using the trapezoidal rule and then performed analyses on the integrals [38].

In vivo values of three photosynthetic parameters: maximum carboxylation capacity (V_c,max_), maximum linear electron transport through photosystem II (J_max_) and respiration in the light (R_d_) were determined by measuring the response of *A *to intercellular [CO_2_] (*Ci*) on August 1 and August 15 2009. *A *vs. *Ci *curves were measured in situ on one young fully expanded leaf of each genotype in all blocks of each treatment (n = 4) with an open gas exchange system (LI-6400, LI-COR, Lincoln, Nebraska). Initially, plants were allowed to reach steady state photosynthesis at their growth [CO_2_] (i.e., 385 ppm or 585 ppm [CO_2_]) at a saturating light level of 1500 μmol m^-2 ^s^-1^. Mean leaf to air vapor pressure deficit (VpdL) was 1.3 ± 0.26 (s.d.), and mean leaf temperature was 26 ± 1°C (s.d.). Once steady state was reached, photosynthetic [CO_2_]uptake rate (*A*) and chlorophyll fluorescence parameters were recorded at the growth [CO_2_]; then [CO_2_] was decreased in 4 or 5 uniform steps to 50 ppm, returned to growth [CO_2_], and then increased in 4 or 5 uniform steps to 1500 ppm [CO_2_]. A minimum of 11 data points were collected for each plant following the methods outlined by Long and Bernacchi [39]. Curves were measured in the morning to avoid confounding treatment and genotype effects with transient decreases in water potential, decreases in chloroplast inorganic phosphate concentration or decreases in maximum photosystem II (PSII) efficiency (Fv'/Fm').

Electron transport rate (ETR), the actual flux of photons driving PSII, and Fv'/Fm' were calculated using fluorescence parameters, Fs, Fm', Fo', [40,41]. Fluorescence parameters were estimated using a Licor 6400 integrated gas exchange system equipped with a fluorescence and light source accessory (LI-6400, LI-COR, Lincoln, Nebraska). Fs is the steady state light adapted fluorescence, Fm' is the maximal fluorescence of a light adapted leaf following a saturating light pulse, and Fo' is the minimal fluorescence of a light adapted leaf that is darkened.

$$\text{ETR}=\left(\frac{Fm^{\prime }-Fs^{\prime }}{Fm}\right)fI\alpha_{leaf}$$

Where *f*, is the fraction of photons absorbed by PSII, assumed be 0.5 for C_3 _plants; I is the incident photon flux density (μmol m^-2 ^s^-1^); and α is leaf absorptance which was constant (0.87).

*A *vs. Ci curves were fitted using a biochemical model of photosynthesis [1] including the temperature response functions determined by Bernacchi et al. [42,43] and were solved for the parameters V_c,max_, J_max _and R_d_. The kinetic constants for Rubisco, Ko, Kc and Γ* in tobacco are taken from [43]. Data below the inflection point of the curve were used to solve for V_c,max _and R_d _using the equation for Rubisco limited photosynthesis [1] and following the method of [39]. Data above the inflection point of the *A *vs. Ci curve were similarly used to solve for J_max _using the equation for RuBP limited photosynthesis [1].

### Leaf traits and final biomass

Leaf disks (ca. 1.9 cm^2^) were collected from plants on August 15 during the midday gas exchange measurements. Leaf disks were sealed in pre-cooled vials, placed in coolers and disk fresh weights were determined the same afternoon. Leaf disks were dried at 60°C for 48 hours and then re-weighed. Dry and wet weights were used to determine specific leaf area (SLA) and specific leaf weight (SLW). These same disks were then ground to a fine powder and used to determine leaf carbon (C) and nitrogen (N) content by total combustion (Costech 4010, Valencia, CA, USA).

Statistical analyses were performed using SAS (Version 9.1, SAS institute, Cary, NC) and Jump (Version 4, SAS Institute, Cary NC). Trait and parameter means of SBPase transformant lines were statistically indistinguishable so the lines were pooled for subsequent ANOVAs. Simple effect tests as implemented in SAS (LSMEANS/SLICE) were used to determine if there were significant differences 1) between types within treatments (i.e., WT ambient vs. SBPase ambient) or 2) between treatments within types (i.e., SBPase ambient vs. SBPase elevated). The diurnals at SoyFACE were analyzed as a repeated measures mixed model analysis of variance (PROC MIXED,SAS). As above, SBPase lines were statistically indistinguishable during the time course and were pooled in ANOVAS. Type (SBPase or WT), CO_2 _concentration [CO_2_] (ambient or elevated), and time of day (time) were fixed factors. Each block contained one ambient and one elevated CO_2 _plot and was considered a random factor. As there were only 4 blocks, significant probability was set at *p *< 0.1 a priori to reduce the possibility of type II errors [44,45].

## Results

### Protein Quantification

**Figure 1:**
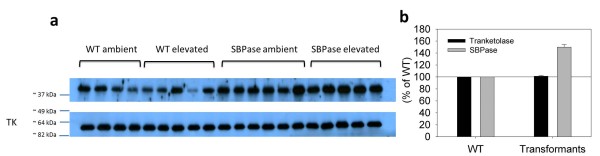
**Western blot and protein quantification for WT and T5 SBPase transformants. **Blots were probed using antibodies raised against SBPase and transketolase. Proteins were detected using horseradish peroxidase conjugated to the secondary antibody. Gels were loaded on an equal protein basis. a) Upper blot is SBPase and the lower is Transketolase (TK) as a loading control. Each lane is a separate individual. b) Quantification for SBPase and TK is based on n = 6 transformants vs. n = 5 WT in ambient CO_2_.

SBPase content was 150% (± 4.5) greater in transformants and more uniform relative to WT plants (Figure 1a and 1b). SBPase overexpressing lines did not differ from each other in terms of the SBPase protein content (Figure 1a). Transketolase content was similar in WT and transformants (Figure 1b).

### Diurnal course of gas exchange and electron transport rate

**Figure 2:**
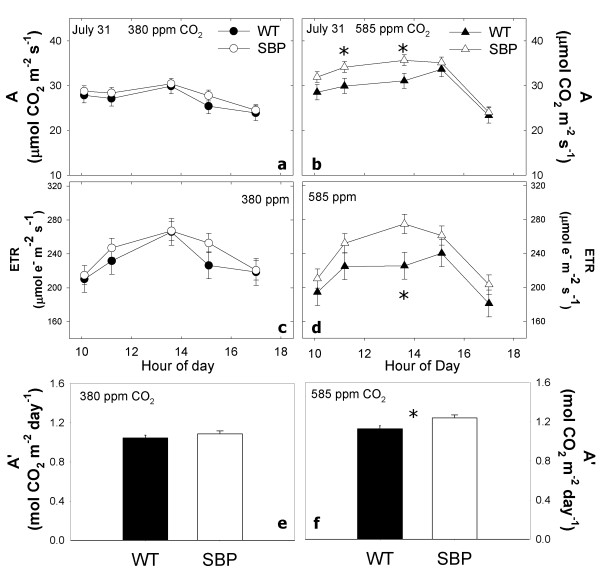
**July 31^st ^diurnal**. Changes in photosynthetic rate (a and b) and electron transport rate (c and d), and the integral diurnal photosynthesis (E and F) for SBP and WT plants grown in the field at ambient (ca. 385 ppm) and elevated CO_2 _(ca. 585 ppm) under fully open air conditions at SoyFACE, Urbana, USA. Symbols are means for n = 3 replicate blocks (± se) for WT and SPBase plants per time point.

**Figure 3:**
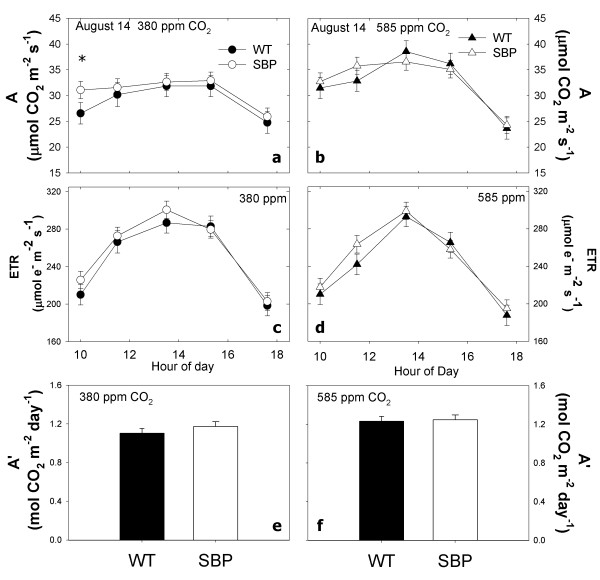
**August 15^th ^diurnal**. Changes in photosynthetic rate (a and b) and electron transport rate (c and d), and the integral diurnal photosynthesis (E and F) for SBP and WT plants grown in the field at ambient (ca. 380 ppm) and elevated CO_2 _under fully open air conditions at SoyFACE, Urbana, USA. Symbols are means for n = 4 replicate blocks (± se) for WT and SPBase plants per time point.

Diurnal trends of photosynthesis and fluorescence parameters were measured at their respective growth [CO_2_] (i.e. 380 or 585 ppm) on July 31 and August 15, 2009 (Table 1). On July 31, photosynthetic rate (*A*) was significantly higher in transformants, due to significant differences around midday at elevated (585 ppm) [CO_2_] (Figure 2a and 2b). On average, electron transport rate (ETR) (Figure 2c and 2d) was significantly higher for transformants at elevated [CO_2_] (simple effect test; F_1,12 _= 8.43 p < 0.05). Differences in ETR between transformants and WT were driven by significantly lower values for WT plants at midday in elevated [CO_2_] on July 31. On August 14, *A *was significantly greater at elevated CO_2 _for both WT and transformants (Figure 3a and 3b, Table 1), however, there were no detectable differences in photosynthesis between WT and transformants. ETR was similar for transformants and WT plants in ambient and elevated CO_2 _on August 14 (Figure 3c and 3d).

**Table 1:** Repeated measures analysis of variance of diurnal variation of photosynthesis (*A*) and linear electron flux through photosystem II (ETR), for the main effects of plant type (tranformants and WT), CO_2 _concentration (385 ppm, 585 ppm), and time of day (time).

31-Jul	Photo					ETR
	*df*	F	*P*	*df*	F	P
type	1, 10.4	10.29	**0.009**	1, 9.11	9.16	**0.014**
CO_2_	1, 10.4	28.93	**0.0003**	1, 9.11	2.04	0.187
type*CO_2_	1, 10.4	1.99	0.188	1, 9.11	1.99	0.191
time	4, 73.7	21.83	**<.0001**	4, 79.9	16.04	**<.0001**
type*time	4, 73.7	0.41	0.804	4, 79.9	0.35	0.846
CO_2_*time	4, 73.7	5.75	**0.000**	4, 79.9	1.58	0.189
type*CO_2_*time	4, 73.7	0.65	0.627	4, 79.9	0.71	0.590

**14-Aug**			**Photo**			**ETR**
	***df***	**F**	***P***	***df***	**F**	**P**

type	1, 12.4	0.98	0.342	1, 10.9	1.54	0.240
CO_2_	1, 12.4	6.58	**0.024**	1, 10.9	2.66	0.131
type*CO_2_	1, 12.4	0.44	0.521	1, 10.9	0	0.971
time	4, 104	29.48	**<.0001**	4, 102	135.52	**<.0001**
type*time	4, 104	0.92	0.453	4, 102	1.16	0.333
CO_2_*time	4, 104	2.73	**0.033**	4, 102	1.64	0.169
type*CO_2_*time	4, 104	0.4	0.806	4, 102	0.45	0.775

Diurnal measurements were collected on July 31 and August 14, 2009.

On July 31, elevating [CO_2_] increased *A' *for WT and transformants (F_1,12 _= 15.93 p < 0.01). Transformants had significantly greater *A' *than WT in elevated [CO_2_] (F_1,12 _= 6.89 p = 0.01), but in ambient [CO_2_] they were not significantly different (compare Figure 2e and 2f). On July 31, *A*' increased 14% for transformants but only 8% for WT. In contrast, on August 15, elevating [CO_2_] increased *A*' by 6% for transformants but by 11% for WT (F1,12 = 6.79 p < 0.05). There were no detectable differences in *A*' between transformants and WT in ambient or elevated [CO_2_] on August 15 (Figure 3e and 3f).

### Photosynthetic biochemical parameters

**Figure 4:**
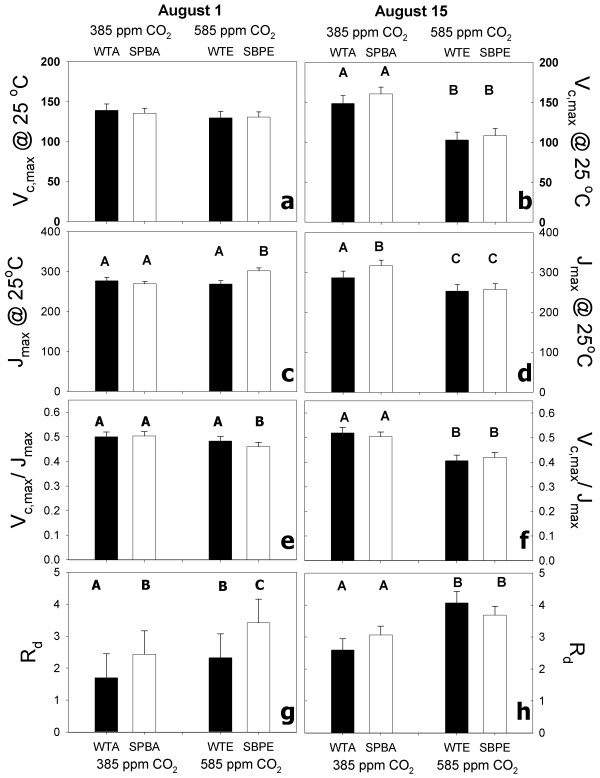
**Photosynthetic parameters derived from response of *A *to [CO_2_] using a biochemical model of photosynthesis (see methods)**. Each day (August 1 and August 15) was analyzed separately with a mixed model ANOVA. Line 11 and line 30 differed only for V/J on aug 1^st ^(*) and were pooled for all other analyses and post hoc tests. Bars are means (± se) (August 1 n = 3) (August 15 n = 4). Bars with different capital letters are significantly different see results for specific p values).

*A *vs. *Ci *curves were measured in the field the morning following each diurnal (i.e. August 1 and August 15) under similar meteorological conditions as the diurnals. On August 1^st ^V_c,max _tended to be lower in elevated [CO_2_] (130.02 ± 5.9) than in ambient [CO_2_] (137.13 ± 5,7) but the trend was not significant (Table 2, Figure 4a). There was a type by [CO_2_] interaction for the response of J_max _(Table 2). Further analysis revealed that growth at elevated [CO_2_] significantly increased J_max _of transformants but not WT (F1,16 = 8.24 p < 0.5)(Figure 4c) on August 1. Consequently, the ratio of V_c,max _to J_max _(V/J) was similar between WT and transformants at ambient [CO_2_]. Elevating [CO_2_] significantly reduced V/J in transformants (F_1,14 _= 15.56 p < 0.01) but not in WT plants on August 1 (Figure 4e). Growth at elevated [CO_2_] significantly increased respiration in the light (R_d_, Table 2) and transformants had significantly higher R_d _than WT in both ambient (F_1,14 _7.78 p < 0.05) and elevated [CO_2_] (F_1,14 _16.03 p < 0.01) (Figure 4g) on August 1.

**Table 2:** ANOVA of photosynthetic paramaters V_c,max @ 2__5_, potential electron transport rate J_max @ 2__5_, V_c,max @ 2__5_/J_max @ 2__5 _(V/J), day respiration (R_d_), for WT and transformants (Type) at ambient and elevated [CO_2_].

1-Aug	Vc,max				Jmax				V/J		Rd	
	*Df*	F	*p*	*df*	F	*p*	*df*	F	*p*	*df*	F	*p*
type	1, 14.2	0.03	0.8661	1, 16	2.58	0.1276	1, 14	1.55	0.2329	1, 14	23.22	**0.0003**
CO_2_	1, 14.2	0.76	0.3979	1, 16	2.44	0.1381	1, 14	5.86	**0.0296**	1, 14	17.87	**0.0008**
type*CO_2_	1, 14.2	0.1	0.7524	1, 16	6.79	**0.0191**	1,14	2.81	0.116	1, 14	0.9	0.3592

**15-Aug**	**Vc,max**				**Jmax**		**V/J**				**Rd**	
	***Df***	**F**	***p***	***df***	**F**	***p***	***df***	**F**	***p***	***df***	**F**	***p***

type	1, 20	2.4	0.1371	1, 20	2.57	**0.1243**	1, 20	0	0.9702	1, 20	0.03	0.8753
CO_2_	1, 20	73.72	**<.0001**	1, 20	18.18	**0.0004**	1, 20	40.21	**<.0001**	1, 20	14.98	**0.001**
type*CO_2_	1, 20	0.3	0.5925	1, 20	1.38	0.2531	1, 20	0.87	0.3608	1, 20	2.5	0.1293

Parameters were derived from *A *vs [CO_2_] curves measured in the field see methods for details. Only three blocks could be measured on August 1.

On August 15, both V_c,max _and J_max _were significantly lower for plants grown under elevated than ambient [CO_2_] (Table 2; Figure 4b and 4d). Transformants had significantly greater J_max _than WT at ambient [CO_2_] but not in elevated [CO_2_] (F_1,20 _= 3.87 p = 0.06). Elevating [CO_2_] significantly decreased V/J in transformants and WT (Table 2 Figure 4f). Elevating [CO_2_] significantly increased R_d _for WT and transformants (Figure 4h).

### Leaf traits and final biomass

**Figure 5:**
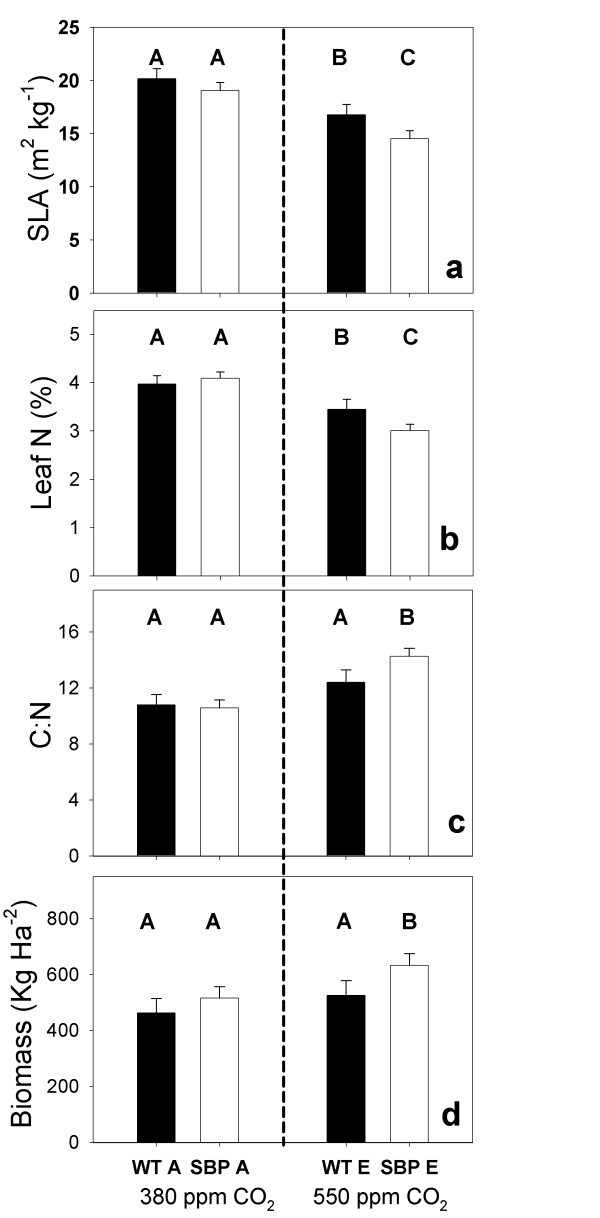
**Plot means for specific leaf area (SLA), leaf nitrogen (N), leaf carbon to nitrogen ratio (C:N), and final above ground biomass for WT and transformants**. Data for SLA, Leaf N and C:N are from the same leaf disks. Therefore leaf N is presented on an equal area basis. Bars with different capital letters are significantly different (see results for specific p values).

Specific leaf area (SLA) was significantly lower at elevated [CO_2_] compared to ambient, and transformants had significantly lower SLA than WT plants (Table 3, Figure 5a). Further analysis revealed that transformant SLA was lower than WT SLA in elevated [CO_2_] (F_1,15 _= 8.75 p < 0.01). Elevating [CO_2_] significantly decreased leaf nitrogen content (%N); consequently, the carbon to nitrogen ratio (C:N) of leaves increased significantly in elevated [CO_2_] (Table 3, Figure 5b and 5c). Transformant C:N increased more than WT (F_1,15 _= 9.46 p = 0.01). Above ground biomass (= yield in kg/Ha) was greater for plants grown in elevated [CO_2_] and transformant biomass was greater than WT plants (Table 3). Biomass increased more for transformants than WT following growth in elevated [CO_2_] (22% vs. 13%) (Figure 5d; F_1,15 _= 6.37 p < 0.05).

**Table 3:** Analysis of variance of the effects of [CO_2_] and plant type (WT vs. Transformant) on specific leaf area (SLA), leaf nitrogen content (%N), leaf carbon to nitrogen ration (C:N) and final biomass (Kg/ha) for n = 3 blocks.

	SLA			%N		C:N		Biomass	
	*df*	F	*p*	F	*p*	F	*p*	F	*p*
type	1,15	6.57	**0.0217**	1.22	0.2875	3.9	**0.0671**	4.05	**0.0625**
CO_2_	1,15	16.69	**0.001**	29.65	**<.0001**	17.36	**0.0008**	5.03	**0.0404**
type*CO_2_	1,15	2.63	0.1257	3.52	**0.0809**	5.65	**0.0312**	0.45	0.5121

## Discussion

The goal of our experiments was to test the hypothesis that tobacco plants transformed to over express the PCR cycle enzyme SBPase would exhibit greater stimulation of carbon assimilation than WT plants when grown at elevated [CO_2_] under field conditions [e.g., [17,30,31]].

### Transformant biomass increases more than WT at elevated [CO_2_]

When grown under fully open air CO_2 _fumigation, SBPase overexpressing plants displayed up to 14% greater light saturated photosynthetic rates (*A*) and up to 21% more linear electron flux through PSII (ETR) than WT plants. Moreover, after 12 weeks of growth at elevated [CO_2_], harvested biomass increased by 13% in WT plants and more than 22% in transformants when compared to plants grown in ambient [CO_2_]. In a prior experiment, the same transformants grown in a greenhouse under prevailing light conditions at ambient [CO_2_](ca. 375 ppm) accumulated 12% more biomass than WT plants (Lefebvre et al. 2005)[16]. Here, at ambient [CO_2_] (ca. 385 ppm) under field conditions, transformants also yielded 12% more biomass than WT plants (see Figure 5) consistent with the Lefebvre et al (2005)[16] greenhouse study. Taken together, these results support our hypothesis and clearly show the benefit of overexpressing SBPase in field grown plants at both current and future levels of atmospheric [CO_2_].

WT biomass was 13% greater in elevated [CO_2_] when compared to ambient grown WT plants, which is somewhat lower than the average increase in biomass for C_3 _crops in FACE experiments [i.e. 19.8% in [46]]. Growth at elevated [CO_2_] alters plant insect interaction and increases palatability of crops [47-50]; thus it is possible that yield stimulations were slightly lower because of aphid and hornworm herbivory (pers obs). In tobacco in particular, aphid infestation significantly reduced the stimulatory effect of [CO_2_] on biomass [51]. Nevertheless, transformant biomass increased more than WT at elevated [CO_2_] (22.7%) and more than the average for C_3 _crops in FACE experiments.

Lefebvre *et al.* (2005)[16] reported that the greatest differences between transformants and WT photosynthetic rates occurred prior to flowering in greenhouse plants and during early development in chamber grown plants. The differences between young expanding and fully expanded leaves could not be accounted for by differential SBPase activity (Lefebvre *et al.* 2005). We show that in ambient and elevated [CO_2_] plots, carbon uptake was enhanced more for transformants during the vegetative phase (i.e. July 31) than when plants were starting to flower (August 15). When plants were beginning to flower, differences between transformants and WT were no longer detectable, yet carbon uptake was consistently stimulated for plants growing in elevated [CO_2_]. Ultimately, even though the realized increase in *A *and *A' *between WT and transformants falls well short of the theoretical 40% increase in assimilation predicted if plants were to reallocate 15% of photosynthetic resources from Rubisco to RuBP regeneration [e.g., [7]], increases in the carbon uptake of transformants early in growth and prior to flowering were sufficiently large to increase final biomass.

Several studies demonstrate that changing expression and activity level of SBPase directly impacts carbon assimilation, growth, and biomass accumulation in tobacco growing at current ambient [CO_2_] (ca. 385 ppm) [16,19,52-55]. While the positive relationship between SBPase activity and carbon assimilation was clearly shown in WT and transformants [16,19], overexpression of SBPase in rice and tobacco has not always increased biomass for plants grown at ambient [CO_2_] levels in controlled environments [16,33,34]. For instance, Lefebvre et al. noted that no increase in photosynthesis or plant yield was evident for tobacco transformants grown in winter when days were shorter and light levels were lower[16] (S. Lefebvre, J.C. Lloyd, and C. Raines unpublished data). The observations of Lefebvre et al. [16] and this study are also consistent with the notion that SBPase exerts control over CO_2 _fixation under light saturating conditions. By definition, the amount of SPBase would not affect the light limited rate of photosynthesis which depends on the rate of production of NADPH and ATP on the photosynthetic membrane. Our diurnal measurements are consistent with these expectations, as transformants with increased SBPase activity showed the greatest increases in carbon assimilation relative to wild type plants around midday when light levels were highest. In contrast, there was no difference in assimilation rates between the SBPase overexpressing and wild type plants at the beginning or end of the day (Figure 2).

### Acclimation to [CO_2_] increases nutrient use efficiency more for transformants than WT

Both WT and transformants showed evidence of a similar decrease in V_c,max _after a month of growth at elevated [CO_2_],indicating photosynthetic acclimation via down regulation of in vivo Rubisco capacity. Photosynthetic acclimation to growth in elevated [CO_2_] is presumed to be a biochemical adjustment to optimize nitrogen use [6]. As [CO_2_] increases so does the catalytic rate of Rubisco, therefore less N needs to be invested in Rubisco to fix carbon. Reallocation of N is then, for instance, available to upregulate respiratory metabolism in response to growth at elevated [CO_2_] [56]. SBPase represents less than 1% of the N contained in the enzymes of photosynthetic carbon metabolism [21]. It is therefore remarkable that ca. 50% increase in the amount of this protein in transformants results in detectable increases in CO_2 _assimilation. The relatively large increase in CO_2 _assimilation at elevated [CO_2_] was associated with a significant decrease in leaf N per unit mass (Figure 5). Thus for a small increase in protein, transformants had a significantly greater increases in nitrogen use efficiency than WT at elevated [CO_2_]. The results are consistent with numerous other FACE studies showing that [CO_2_]will stimulate growth in spite of photosynthetic acclimation and that growth at elevated [CO_2_]increases nitrogen use efficiency [reviewed in [57]].

Transformants and WT plants grown in elevated [CO_2_] tended to have higher respiration in the light (R_d_) than plants in ambient [CO_2_]plots. Leaves of plants grown under elevated [CO_2_] accumulate larger concentrations of non-structural carbohydrates (i.e. sugar and starch) [46], and this may underlie higher respiration [58]. Recently, Leakey et al. [56] demonstrated that the acclimation response of respiration to elevated [CO_2_] was mediated via transcriptional upregulation of respiratory enzymes. We speculate that the reportedly greater sucrose and starch accumulation in transformants [16] stimulates additional acclimation of respiration to elevated [CO_2_] and may therefore also diminish the benefit of overexpressing SBPase. Alternatively, higher R_d _in transformants may be a result of the unregulated overexpression of the enzyme. Either way, higher R_d_, the requirement for high light, and unmeasured natural stresses all would contribute to a lower realized benefit to overexpressing SBPase in the field.

## Conclusion

The data presented in this paper have demonstrated that transgenic tobacco plants with increased SBPase have the potential for greater stimulation of photosynthesis and biomass production relative to wild type tobacco when grown at elevated [CO_2_]. Differences between theoretical and realized increases in carbon assimilation are to be expected as studies of PCR cycle antisense plants have demonstrated that the relative importance of any one PCR cycle enzyme is not fixed and will vary according to environmental and developmental conditions [[20], this study,[59]]. Nevertheless, our findings are consistent with the notion that elevating [CO_2_] increases the metabolic control of RuBP-regeneration and decreases the control exerted by Rubisco at light saturation [6,7]. Though smaller than theoretically predicted, the increases in photosynthetic stimulation at elevated [CO_2_] demonstrated here are indicative that C_3 _crop plants can be engineered to meet a rapidly changing environment.

## Authors' contributions

DR Conceived and designed the experiment, acquired and analyzed the data, and wrote the paper. AL aided in data acquisition and analysis, revised the paper, and gave final approval of the manuscript. MK aided in data acquisition, data analysis and gave final approval of the manuscript. CR provided the transformants, provided technical support, revised the paper, and gave final approval of the manuscript. SL and DO conceived and aided in the design of the experiment, revised the manuscript, and gave final approval of the manuscript.
